# Injection Techniques to Reduce Adverse Effects of Subcutaneous Low‐Molecular‐Weight Heparin Among Patients With Cardiovascular Diseases: A Scoping Review

**DOI:** 10.1111/jan.16475

**Published:** 2024-09-25

**Authors:** Arkers Kwan Ching Wong, Rachel Yui Ki Chu, Ying Nan, Huilin Cheng, Danny Tong, Ming Leung, Harris Lam, Sin Hing Chiu, Heung Wan Cheung, Miu Ching Chan, Mei Yi Chau, Terence Lee, Yuen Wa Leung, Hoi Ching Mow, Sylvia Wan, Lee Yuen Wong, Jed Montarye

**Affiliations:** ^1^ School of Nursing The Hong Kong Polytechnic University Kowloon Hong Kong SAR; ^2^ School of Nursing Southern Medical University Guangdong China; ^3^ Nursing Services Department Hospital Authority Head Office Kowloon Hong Kong SAR; ^4^ Department of Medicine and Geriatrics Kowloon West Cluster Kowloon Hong Kong SAR; ^5^ Department of Medicine and Therapeutic New Territories East Cluster New Territories Hong Kong SAR; ^6^ Department of Medicine and Geriatrics Kowloon Central Cluster Kowloon Hong Kong SAR; ^7^ Department of Medicine and Geriatrics Hong Kong West Cluster Hong Kong Island Hong Kong SAR; ^8^ Department of Medicine and Geriatrics Hong Kong East Cluster Hong Kong Island Hong Kong SAR; ^9^ Department of Medicine & Geriatrics Kowloon East Cluster Kowloon Hong Kong SAR; ^10^ Department of Medicine and Geriatrics New Territories West Cluster New Territories Hong Kong SAR; ^11^ Department of Cardiothoracic Surgery Hong Kong West Cluster Hong Kong Island Hong Kong SAR

**Keywords:** cardiovascular diseases, low‐molecular‐weight heparin, scoping review, subcutaneous injections

## Abstract

**Aim(s):**

To systematically review the existing literature and address the following research question: What are the most effective techniques used to minimise adverse effects resulting from subcutaneous injections of low‐molecular‐weight heparin among patients with cardiovascular diseases?

**Design:**

A scoping review.

**Methods:**

A comprehensive search was conducted across multiple databases, including CINAHL, PubMed, EMBASE and the Cochrane Library, from 1 February 2014 to 31 January 2024. Participants were aged 18 years or older, diagnosed with venous thromboembolism or arterial thromboembolism and had prescribed subcutaneous injections of low‐molecular‐weight heparin. The collected data were analysed following the Joanna Briggs Institute approach, and it was organised and categorised based on the main objectives of the review.

**Results:**

Twenty studies were eligible, including 1 best practice project, 7 randomised controlled trials and 9 quasi‐experimental studies. The techniques under investigation encompassed various aspects, including the injection site, injection duration (e.g., 30 s vs. 10 s), injection method (e.g., needle insertion angle), duration of needle withdrawal after injection, pressure application time and cold pressure. Preliminary evidence suggests that techniques such as using the abdominal site and slower injection rates may help reduce adverse effects. However, the optimal parameters for injection duration, waiting time, pressure and cold application, including the duration of these applications, remain uncertain due to limitations in sample size and heterogeneity in interventions and outcome measures across the studies.

**Conclusions:**

Ensuring the accurate administration of low‐molecular‐weight heparin is of utmost importance as it plays a critical role in decreasing mortality rates and minimising substantial healthcare costs linked to complications arising from incorrect administration. The findings from the current review have significantly contributed to strengthening the evidence base in this field, providing more robust and reliable information.

**Implications for the Profession:**

This review emphasises the significance of implementing standardised subcutaneous injection techniques for low‐molecular‐weight heparin in patients with cardiovascular disease in order to reduce complications and enhance patient outcomes.

**Reporting Method:**

This study followed the applicable guidelines established by the PRISMA 2020 statement. The PRISMA checklist for systematic reviews was utilised for reporting purposes.

**Patient or Public Contribution:**

There is no patient or public contribution to declare.

**Trial Registration:**

OSF registries: osf.io/phk72

**Summary**

- What Problem did the Study Address?
  ○ This scoping review synthesizes current evidence around the various injection techniques, guiding healthcare professionals to minimize adverse effects associated with low‐molecular‐weight heparin administration in cardiovascular disease patients.
- What were the Main Findings?
  ○ The findings encourage the standardization of low‐molecular‐weight heparin subcutaneous injection procedures, potentially leading to a decrease in complications such as bruising and bleeding, thus enhancing patient safety and comfort.
- Where and on Whom will the Research have an Impact?
  ○ Stimulate exploration of optimal low‐molecular‐weight heparin injection parameters, aiding in the development of comprehensive guidelines for practice worldwide.

## 1 Introduction

Cardiovascular diseases (CVDs) have collectively remained the leading cause of global mortality and a significant contributor to disability (World Heart Report 2023). More than half a billion people around the world continue to be affected by CVDs, accounting for 20.5 million deaths in 2021 (Lindstrom et al. 2022). Among various CVDs, venous thromboembolism (VTE), such as deep vein thrombosis and pulmonary embolism, and arterial thromboembolism (ATE) in myocardial infarction and ischaemic stroke significantly cause mortality in many countries, which is about one in four deaths worldwide (Ma et al. 2024; Wendelboe and Weitz 2024). Additionally, it has been suggested that one‐third of patients with VTE are at risk of recurrence and serious long‐term complications, including postthrombotic syndrome and venous ulcers (Mensah et al. 2015). These disorders not only significantly impact the quality of life of patients but also impose a substantial burden on healthcare costs and society as a whole (Duffett, Castellucci, and Forgie 2020; Lutsey and Zakai 2023).

## 2 The Review

Low‐molecular‐weight heparin (LMWH) is commonly prescribed for preventing or treating thrombus formation and associated complications for patients with CVDs (Chung 2016; Ortel et al. 2020; Stevens et al. 2021). However, the improper administration of subcutaneous LMWH can lead to adverse effects like bruising, haematoma, induration and pain at the injection site (Andras, Sala Tenna, and Stewart 2017; Li et al. 2021). Although rare, potentially life‐threatening complications such as retroperitoneal and intra‐abdominal haematoma can occur, which are frequently misdiagnosed and associated with a higher mortality rate (Yang et al. 2020). Research suggests that standardising subcutaneous injection procedures, including factors such as injection site, speed, angle and pressure time, should be considered in order to reduce the incidence of related complications (Fidan, Şanlialp Zeyrek, and Arslan 2023).

Although previous studies have examined interventions to mitigate the adverse effects of LMWH injections, there is a notable lack of a comprehensive synthesis of this growing body of evidence. Additionally, it is important to note that there is a lack of detailed procedural guidance regarding the optimal administration of LMWH via the subcutaneous route. For instance, uncertainties exist regarding the ideal injection duration, the appropriate wait time before needle withdrawal, the use of airlock techniques and the duration of pressure application. The absence of comprehensive best practice protocols presents challenges in terms of education, quality improvement and maintaining consistent clinical practice.

## 3 Aim(s)

In light of these considerations, the objective of this scoping review is to methodologically identify and categorise the available literature on techniques aimed at mitigating adverse effects associated with subcutaneous LMWH injections for CVDs. Through the consolidation of reported interventions, outcomes and existing knowledge, this review aims to provide guidance for future research endeavours, establish an evidence base for guideline development and facilitate the implementation of educational and quality improvement initiatives in clinical settings. The long‐term goal is to enhance patient safety, improve the overall experience and optimise outcomes for the substantial population necessitating frequent LMWH injections.

## 4 Methods

The study followed a systematic scoping review methodology, adhering to the PRISMA Checklist (Page et al. 2021; Tricco et al. 2018). The objective was to identify evidence‐based recommendations and guidelines for administering subcutaneous LMWH injections in patients with CVDs (OSF registration reference no.: osf.io/phk72).

### 4.1 Eligibility Criteria

The eligibility criteria for the selection of studies, guidelines and other relevant sources of information were included if they provided empirical data on the subcutaneous injection technique of LMWH to minimise adverse effects in CVDs. The eligible sources of information covered recommendations, guidelines, clinical trials and observational studies conducted in any location and reported in English. Simulated studies and studies not specifically related to cardiac patients were excluded.

### 4.2 Information Sources and Search Strategy

We conducted a comprehensive search including the following databases from 1 February 2014 to 31 January 2024: CINAHL, PubMed, EMBASE and the Cochrane Library databases. To ensure the thoroughness of our search, a manual search was performed by reviewing the reference lists of the included studies, grey literature and guidelines to identify additional relevant sources. The search strategy adopted a combination of specific search terms and medical subject headings (MeSH) to ensure relevant results. Taking PubMed as an example, the search type is (‘heparin’[Mesh]/‘low‐molecular‐weight heparin’ [Mesh]/‘anticoagulant’[Mesh]) AND (‘injection’[Mesh]/‘subcutaneous injection’ [Mesh]/‘administration’ [Ti/Ab]/’injection technique’[Mesh]) AND (‘cardiac’ [Ti/Ab]/’heart disease’ [Mesh]) AND (‘adverse effects’[Mesh]/‘complications’ [Mesh]).

### 4.3 Selection of Sources

**FIGURE 1:**
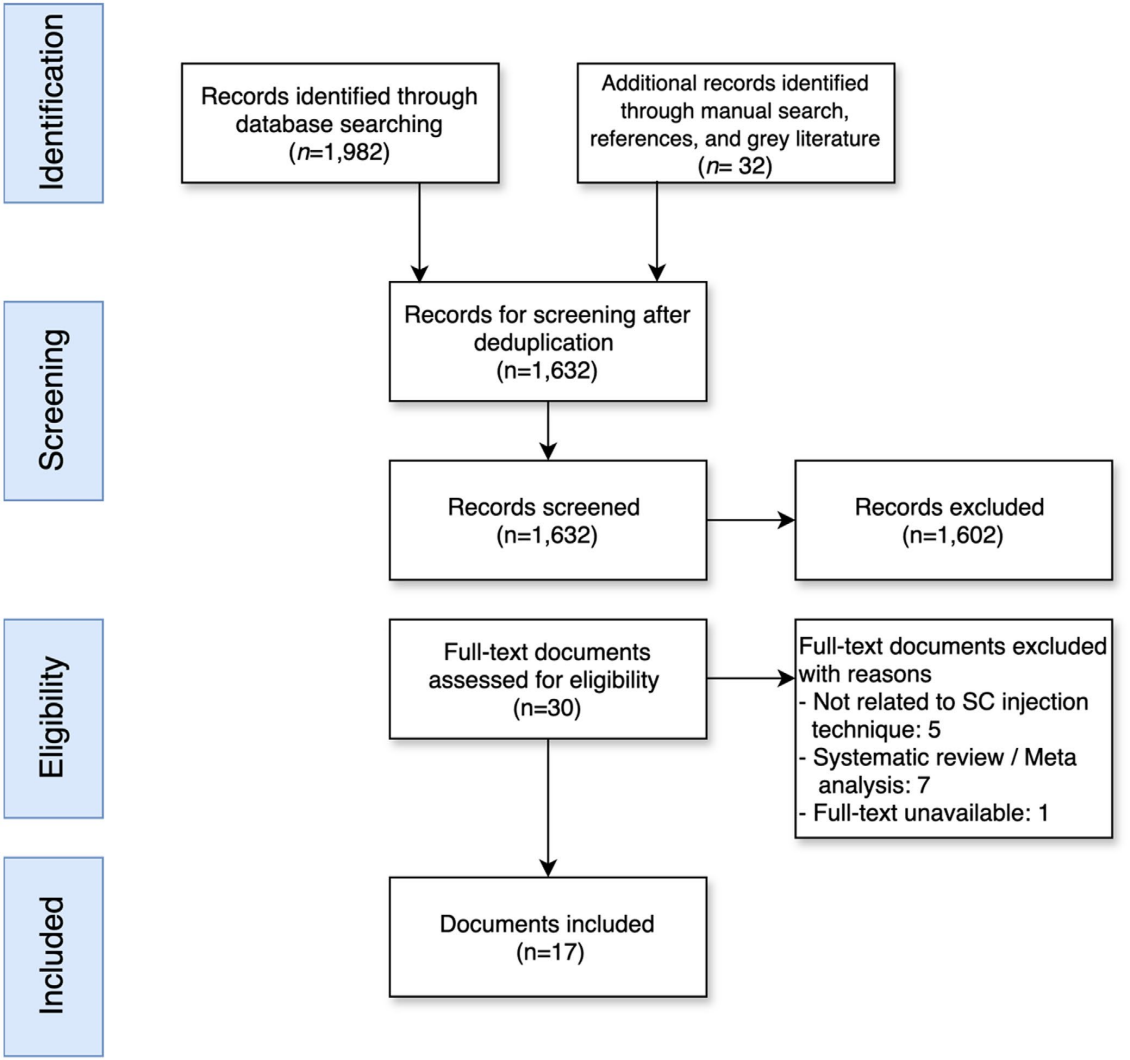
Flowchart of the study.

Two reviewers (Y.N. and R.Y.K.C.) independently screened the titles and abstracts of the retrieved articles to identify potentially relevant studies. Full‐text articles were assessed for eligibility based on predefined inclusion and exclusion criteria. Discrepancies between the reviewers were resolved through thorough discussion and consensus, involving the participation of the third author (A.K.C.W.). Data from the included studies and guidelines were extracted using a standardised form. The extracted data included study characteristics (e.g., author, year, and study design), patient characteristics (e.g., sample size), details of the subcutaneous injection technique (e.g., injection site and injection duration) and reported adverse effects. The search and selection process are summarised in the flow chart (Figure 1).

### 4.4 Data Extraction and Data Analysis

A narrative synthesis was conducted to summarise the findings from the included studies and guidelines. The results were organised according to the various aspects of the subcutaneous injection technique, such as injection site, injection duration and pressing time. Recommendations and best practices were derived from the synthesis of the evidence and presented in a clear and concise manner. The collected data were analysed using the Joanna Briggs Institute (JBI) approach. This systematic methodology involves comprehensively reviewing existing literature, synthesising the evidence, critically appraising the studies and developing evidence‐based guidelines or recommendations for healthcare practice (Munn et al. 2020). The data were then organised and categorised based on the primary objectives of the review. The findings are presented in a table format along with a narrative summary to provide a comprehensive overview.

## 5 Results

### 5.1 Results of the Search

The initial database search yielded 1982 records, and an additional 32 records were identified through other sources, including reference lists of included documents. After removing duplicate records and conducting a screening of titles and abstracts, 1632 documents were obtained in full for further evaluation. A total of 31 papers were assessed for eligibility after obtaining the full‐text documents. Among these, 13 documents were subsequently excluded based on predefined inclusion criteria. Eventually, a total of 17 documents were included in the review, as depicted in Figure 1.

### 5.2 Study Characteristics

Among the 17 studies, a total of 1752 participants were included, ranging from 30 to 260. All participants were aged 18 years or above. These studies were conducted in various countries between 2014 and 2024. The majority of the studies and their participants were from Iran (*n* = 6) and Turkey (*n* = 4), while the remaining studies included participants from various countries such as India (*n* = 2), China (*n* = 1), Spain (*n* = 1), Singapore (*n* = 1), Thailand (*n* = 1) and Middle Eastern country (*n* = 1). The characteristics of the included studies were extracted and are summarised in Table 1.

**TABLE 1:** Characteristics of included studies.

Phases	Document	Country	Sample	Design	Study aim	Intervention	Key findings
Preinjection	Injection sites (*n* = 4)						
	Pourghaznein, Azimi, and Jafarabadi (2014)	Iran	90	A quasi‐experimental study	To compare the effects of four methods of subcutaneous heparin injection on pain and bruising in abdomen and thighs	Abdomen versus thighs	The severity of pain in abdomen was lower than in thighs
	Jareño‐Collado et al. (2018)	Spain	301	A randomised controlled trial	To evaluate the adverse events, ecchymosis and/or haematoma after the administration of prophylactic subcutaneous enoxaparin in the abdomen versus the arm in the critically ill patient	The anterolateral and posterolateral abdominal waist versus the rear face of the arm	Prophylactic subcutaneous enoxaparin administered in the abdomen causes fewer haematomas after 72 h, than when administered in the arm
	Babaieasl et al. (2018)	Iran	40	A quasi‐experimental study	To compare adverse effects when LMWH was administered at two different sites	The lateral abdomen is about 5 cm from the umbilicus versus deltoid	Deltoid is a more appropriate injection site for LMWH than the abdomen to minimise bruising
	Sarani et al. (2020)	Iran	60	A randomised controlled trial	To compare the effect of duration and site of subcutaneous injection of enoxaparin on pain intensity and bruise size in patients admitted to cardiac care units	Abdomen versus arm	Abdomen is recommended for subcutaneous injection of enoxaparin
During inoculation	Injection duration (*n* = 5)						
	Dehghani, Najari, and Dehghani (2014)	Iran	70	A quasi‐experimental design	To define the effect of sodium enoxaparin subcutaneous injection on the bruise size	Methods A: 10 s; Methods B: 30 s	Length of enoxaparin subcutaneous injection has no effect on the bruising size
	Dadaeen et al. (2015)	Iran	100	A randomised controlled trial	To determine the effect of duration of subcutaneous injection of enoxaparin sodium on the extent of bruising and pain intensity at the patients' injection sites in 2013	Methods A: 10 s; Methods B: 30 s	30 s injection significantly less than 10 s injection
	Ahmadi et al. (2016)	Iran	86	A quasi‐experimental study	To assess the effect of increasing the heparin injection time on pain intensity and bruising associated with subcutaneous injection	Methods A: 10 s; Methods B: 30 s	By elevating the duration of heparin injection, the severity of pain was reduced
	Bijani et al. (2016)	Iran	70	A quasi‐experimental study	To compare the duration of subcutaneous injection of Clexane (enoxaparin) on the bruising	Methods A: 10 s; Methods B: 30 s	The subcutaneous injection duration be increased to 30 s to enhance the quality of care and minimise the unpleasant and stressful experience for patients
	Kattunilam and Rohini (2016)	India	30	A quasi‐experimental study	To assess the effect of time duration in injecting subcutaneous LMWH on pain and bruising among patients with myocardial infarction (MI)	Methods A: 10 s; Methods B: 30 s.	Slow injection of LMWH over 30 s helps in reducing pain and occurrence of bruises at the injection site
	Technique (*n* = 2)						
	Geng, Zhang, and Shi (2018)	China	260	A randomised controlled trial	To analyse the clinical values of modified injection of LMWH in reducing subcutaneous bleeding and pain	Methods A: angle of 30° ~ 40°; the injection site was compressed with a cotton swab for 2 ~ 3 min; Methods B: inserted vertically; the skin was pinched up continuously for 3 to 5 min; the needle insertion site was not compressed	Modified low‐molecular‐weight heparin injection can effectively reduce the incidence of bleeding and pain, which is beneficial to the compliance and quality of life of elder patients
	Neo, Seow, and Tho (2021)	Singapore	Three adult cardiology and cardiothoracic general wards	Best practice implementation project	To utilise an evidence implementation framework to introduce evidence‐based injection techniques, for the reduction of postinjection complications	Introduction of a new subcutaneous injection workflow based on evidence‐based techniques; 1. Subcutaneous injection is performed at a 90° angle to the skin using the pinch‐up technique (or if appropriate, 45° angle without the pinch‐up technique). 2. The medication is injected over a 10 s duration 3. Following release of medication, a 10 s wait is observed prior to the removal of the needle	1. The incidence of bruising reduced from baseline, with a relative risk reduction of 52% (1 month) and 29% (8 months); 2. Median pain also decreased from the baseline, with an improvement from 2.0 (1.0–3.0) to 0.0 (0.0–1.0)
	Injection duration and duration of needle withdrawal after injection (*n* = 3)						
	Pourghaznein, Azimi, and Jafarabadi (2014)	Iran	90	A quasi‐experimental study	To determine and compare the effects of four methods of subcutaneous heparin injection on pain and bruising in abdomen and thighs	Method A: 10 s injection duration; Method B: 10 s injection duration and waiting for 10 s before withdrawing the needle; Method C: 15 s injection duration and waiting for 5 s before withdrawing the needle; Method D: 5 s injection duration and waiting for 15 s before withdrawing the needle	The method 15 s injection duration and waiting for 5 s before withdrawing the needle is recommended to be used for subcutaneous heparin injection by clinical nurses
	Jueakaew et al. (2019)	Thailand	44	A randomised controlled trial	To investigate the efficacy of a novel LMWH injection technique compared to the standard technique relative to bruising incidence, bruise size and pain	Methods A: 10 s injection duration with immediate needle withdrawal; Methods B: a 30 s injection duration with a 10 s pause before needle withdrawal	A 30 s duration injection with a 10 s pause before needle withdrawal resulted in significantly fewer and smaller bruises
	Sarani et al. (2020)	Iran	60	A randomised controlled trial	To compare the effect of duration and site of subcutaneous injection of enoxaparin on pain intensity and bruise size in patients admitted to cardiac care units	Methods A: 30 s duration injection with immediate needle withdrawal; Methods B: 15 s injection duration and waiting for 5 s before withdrawing the needle	The 30 s injection technique resulted in a significant decrease in pain intensity, bruise size and bruising extent due to the subcutaneous injection of enoxaparin
Postinjection	Pressing time (*n* = 2)						
	Çi̇t and Şenturan (2018)	Turkey	49	A quasi‐experimental study	To examine the effect of applying pressure for a minute on bruising in subcutaneous heparin injection	Methods A (control practice): applied for 3 or 4 s; Methods B (experimental practice): applied for 60 s	Applying pressure on the injection area for a minute following a subcutaneous heparin injection reduced the development rate of bruising
	Karabey and Karagözoğlu (2021)	Turkey	100	A quasi‐experimental design	To determine the effect of manual pressure application on pain and comfort level in SC injection	Methods A: standard application; Methods B: manual pressure	Manual pressure application is a more effective method in reducing pain due to subcutaneous injection compared to the standard application
	Cold application (*n* = 3)						
	Amaniyan et al. (2016)	Middle Eastern country	180	A randomised controlled trial	To assess the effect of the application of local cold and cold–hot packs upon the size of bruising at the injection site of subcutaneous enoxaparin sodium	Methods A: local cold gel pack group; Methods B: local cold–hot gel pack group; Methods C: a control group who received no applications of packs	The cold–hot pack group had significantly less (in number) and smaller (in size) injection site bruising than the two other groups at 48 and 72 h
	Varghese (2022)	India	50	A quasi‐experimental study	To determine how cold application affected ICU patients' pain and bruises caused by subcutaneous injection of LMWH	Cold application was given for 3 min prior to and 5 min after the administration of subcutaneous injection of enoxaparin	In clinical practice, a cold application utilising frozen gel packs for 3 min before and 5 min after the injection of LMWH is effective
	Karadağ et al. (2023)	Turkey	72	A randomised controlled trial	To determine the effect of cold application and compression on pain and bruising in subcutaneous heparin injection	Methods A: pressure for 60 s; Methods B: cold application; Methods C: no procedure	The compression group had smaller bruising size and lower pain in contrast with the other groups

### 5.3 Methodological Aspects of the Studies

Of the 17 articles included in this scoping review, one was best practice implementation project, seven studies were randomised controlled trials (RCTs) and the remaining studies were quasi‐experimental studies.

All 17 studies employed quantitative data analysis methods, utilising various validated measurement tools and instruments to assess pain. These included established questionnaires such as the McGill Pain Questionnaire (Campbell, Johnson, and Zernicke 2013), standardised scales like the Verbal Pain Scale developed by Melzack and Katz (2013) and widely used measurement instruments like the Numerical Rating Scale (NRS) (Thong et al. 2018) and the Visual Analog Scale (VAS) (Thong et al. 2018). Additionally, researchers also employed precise measurement tools such as flexible plastic rulers, the VISITRAK Digital Wound Assessment System (Sugama et al. 2007) and comfort scores to capture relevant pain‐related data in a rigorous and systematic manner.

### 5.4 Programme Content

Based on the injection process, the literature included in this scoping review can be classified into three distinct phases: preinjection, during injection and postinjection. During the preinjection phase, four studies focused on the injection site. In the injection phase, the most prominent aspect was injection duration time, with five studies dedicated to exploring its effects, followed by two studies that examined the method of injection. Additionally, three studies provided insights into both the duration of injection and the wait time before needle withdrawal, underlining their combined importance in the injection process. Transitioning to the postinjection phase, two studies investigated the effects of cold application after injection and other three studies specifically looked at the impact of pressing time.

#### 5.4.1 Injection Site

Among the four studies related to injection sites, three studies compared abdominal injections with injections in the arm deltoid muscle and the remaining one study compared abdominal injections with thigh injections. The findings from these studies were varied. Three studies indicated that the abdomen is a preferable injection site, while one study suggested that the deltoid muscle is a more appropriate injection site for LMWH to minimise bruising. Specifically, RCTs by Jareño‐Collado et al. (2018) between July 2014 and January 2017 in an intensive care unit found that prophylactic subcutaneous enoxaparin administered in the abdomen resulted in fewer haematomas after 72 h compared to administration in the arm (*p* = 0.027). Pourghaznein et al. (2014) compared abdomen and thigh sites and found significantly higher pain severity in the thigh but no significant difference in bruising size or number between the sites. Additionally, Sarani et al. (2020) in a 2019 study of 60 cardiac patients aged 42–75 years observed that the mean bruise size at 48 h was significantly smaller in the abdomen compared to the arm (*p* < 0.001). Conversely, Babaieasl et al. (2018) involved 40 patients in a coronary care unit who participated in a within‐subject study where injections were administered at the lateral abdomen and deltoid. The results showed a significant difference in bruising size between the two injection sites after 72 h (*p* = 0.02). It is suggested that the deltoid is a more appropriate injection site for LMWH than the abdomen to minimise bruising (Babaieasl et al. 2018).

In summary, limited evidence suggests the abdomen may be associated with less bruising compared to other sites like the arm and thigh for LMWH injection, but more research is needed on optimal injection locations.

#### 5.4.2 Injection Duration

The duration of the injection was categorised into fast and slow injections. Among the five articles reviewed, a fast injection was defined as lasting 10 s, while a slow injection was characterised as taking 30 s. Of the reviewed articles, 4 of them reported that slow injection significantly reduced pain intensity and bruising. However, one article concluded that injection duration was not associated with the occurrence of pain or bruising. Ahmadi et al. (2016) reported significantly lower pain intensity and bruising rates with 30 s injections compared to 10 s injections in individuals aged 40–69 (*p* = 0.005). Bijani et al. (2016) showed higher bruising rates at 48 h (10 s: 21.7 ± 16 vs. 30 s: 14.24 ± 2.38) and 60 h (10 s: 15.65 ± 10.67 vs. 30 s: 12.98 ± 8.12) after 30 s compared to 10 s injections (*p* < 0.05). Kattunilam and Rohini (2016) involved 30 patients who received LMWH while being admitted with myocardial infarction. The study found that the mean pain score (45.71) during the standard technique (10 s) was greater than the mean pain score (25.38) during the modified technique (30 s) (*p* < 0.05). Dadaeen et al. (2015) applied a randomised cross‐over design and found significantly lower bruising and pain with 30 s versus 10 s injection (mean extent of bruising after 48 and 72 h: 30 s: 23.69 ± 3.27 mm^2^ and 14.76 ± 3.52 mm^2^; 10 s: 45.53 ± 6.35 mm^2^ and 26.45 ± 4.70 mm^2^; *p* < 0.001) (pain intensity scores: 30 s: 0–8; 10 s: 2–10; *p* < 0.001).

However, in the study conducted by Dehghani, Najari, and Dehghani (2014) with patients aged between 35 and 75, no significant difference was observed in bruise sizes between the 10 and 30 s injection durations. Overall, moderate evidence supports possible benefits of longer injection duration, but some inconsistencies exist.

#### 5.4.3 Injection Duration and Duration of Needle Withdrawal After Injection

Departing from the earlier emphasis on injection duration alone, subsequent studies illuminate the synergistic effects of varying both injection duration and pause times before needle withdrawal. However, it is important to note that the time intervals examined in these studies vary. In an RCT with 44 DVT patients by Jueakaew et al. (2019), two groups were compared: one with a 10 s injection followed by immediate withdrawal, and another with a 30 s injection plus a 10 s pause before withdrawal. The latter group showed a significantly smaller mean bruise size (*p* < 0.05). Sarani et al. (2020) conducted a study with 60 cardiac patients, comparing a 30 s injection with immediate withdrawal against a 15 s injection with a 5 s pause. The former group significantly reduced pain and bruising at 24 and 48 h postinjection (*p* < 0.001). Pourghaznein, Azimi, and Jafarabadi (2014) involving 90 hospitalised patients, four injection methods were compared: Method A (10 s injection duration), Method B (10 s injection duration and waiting for 10 s before withdrawing the needle), Method C (15 s injection duration and waiting for 5 s before withdrawing the needle) and Method D (5 s injection duration and waiting for 15 s before withdrawing the needle). The study found that Method C resulted in a significantly lower number of bruises and smaller bruise size compared to the other methods (*p* < 0.05).

#### 5.4.4 Injection Technique

In studies examining different injection techniques, Neo, Seow, and Tho (2021) conducted a best practice implementation project to improve subcutaneous injection techniques based on the hospital's standard operating protocol. They introduced three criteria: (1) injecting at a 90° angle using the pinch‐up technique or 45° angle when appropriate; (2) administering the medication over 10 s; and (3) waiting for 10 s before removing the needle. These techniques resulted in lower rates of bruising and pain associated with the injections. Geng, Zhang, and Shi (2018) studied 260 patients, comparing conventional injections (angle of 30° ~ 40°; the injection site was compressed with a cotton swab for 2 ~ 3 min) with modified techniques (inserted vertically; the skin was pinched up continuously for 3–5 min; the needle insertion site was not compressed) finding that modified injections effectively reduced bleeding and pain, improving patient compliance and quality of life.

#### 5.4.5 Pressure Duration

In the category of duration of pressure application after injection, Çi̇t and Şenturan (2018) conducted a quasi‐experimental study with 49 patients, comparing 3 s versus 60 s of pressure application. They found significantly less bruising at 24, 48 and 72 h with 60 s of pressure (*p* < 0.05). Karabey and Karagözoğlu (2021) in a study with 100 internal medicine patients aged 18–65 years, compared no manual pressure against 10 s of manual pressure, finding that manual pressure notably reduced pain and improved comfort (*p* = 0.001).

Overall, moderate evidence on duration of pressure application supports possible benefits of longer pressure, but the optimal duration remains uncertain. Further research is needed to determine the optimal duration of pressure to apply after injection.

#### 5.4.6 Cold Application

In studies exploring cold application postinjection, Amaniyan et al. (2016) randomly allocated 180 coronary disease patients into three groups: (i) local cold gel pack group, (ii) local cold–hot gel pack group and (iii) a control group with no pack applications. The result suggested that cold–hot pack group exhibited significantly fewer and smaller injection site bruising compared to the other two groups at 48 and 72 h (*p* < 0.001). Varghese (2022) reported that in 50 ICU patients prescribed LMWH, a 3 min preinjection and 5 min postinjection cold application using frozen gel packs effectively reduced injection site issues.

However, the effects of a 60 s pressure application, cold application and no procedure were compared in RCTs conducted by Karadağ et al. (2023) in Turkey, involving 72 patients with COPD (Chronic Obstructive Pulmonary Disorder). The results showed that the compression group had smaller bruising size and lower pain compared to the other groups.

## 6 Discussion

### 6.1 Summary of Evidence

Optimising the subcutaneous injection technique for administering LMWH is vital in improving patient outcomes and ensuring the safe and effective delivery of medication. In this discussion, we will explore the implications of various injection techniques, including factors such as injection sites, injection durations, waiting times before needle withdrawal and postinjection care practices, on clinical practice.

#### 6.1.1 Injection Site Selection

The abdominal region has been widely recognised as the preferred injection site for LMWH administration due to several advantages (Jareño‐Collado et al. 2018; Pourghaznein, Azimi, and Jafarabadi 2014; Sarani et al. 2020). The presence of abundant subcutaneous adipose tissue, along with lax skin and a lower density of nerve fibres, reduces the likelihood of intramuscular injections, thereby minimising pain and associated complications (Kawakami, Ishihara, and Mihara 2001; Lancerotto et al. 2011; Pirri et al. 2023). The larger injection area and rich capillary network in the abdomen contribute to optimal drug absorption, enhancing the efficacy of LMWH (Nakamura et al. 2008). Additionally, the abdominal region offers convenient accessibility, making it particularly suitable for self‐administration by patients. The lower risk of bleeding compared to peripheral joints in the limbs further supports the choice of choosing abdomen as the preferred injection site. A meta‐analysis by Li et al. (2021) found that arm injections were associated with a significantly higher risk of bruising compared to abdominal injections (RR: 0.76; 95% CI: 0.64–0.90). Patients injected in the abdomen also reported less pain than those injected in the arm (RR: 0.57; 95% CI: 0.48–0.67). Further supporting this, Jareño‐Collado et al. (2018) reported a lower incidence of observed ecchymosis in the abdomen (44.3%) compared to the arm (51.1%). The incidence of haematoma was also lower in the abdomen (6%) than in the arm (9.9%). Notably, all haematomas on the abdomen were 2 mm^2^ or less, while those on the arm reached up to 20 mm^2^.

#### 6.1.2 Injection Duration and Waiting Time

Studies suggest that slower injection durations, such as extending the injection time from 10 s to 30 s, have shown benefits in terms of reducing pain intensity and minimising bruising (Ahmadi et al. 2016; Bijani et al. 2016; Dadaeen et al. 2015; Kattunilam and Rohini 2016). Slowing down the injection speed allows for more time for tissue absorption, thereby reducing local drug concentration and minimising tissue stimulation (Kim, Park, and Lee 2017; Richter, Bhansali, and Morris 2012). This approach not only helps mitigate tissue damage and injury associated with faster injection techniques (Hall and Hall 2020; Lee and White 1913) but also reduce drug accumulation at the injection site and alleviate tissue pressure.

#### 6.1.3 Postinjection Care Practices

Additional techniques, such as the airlock technique and cold application, have been investigated to further mitigate bruising and pain resulting from LMWH injections (Amaniyan et al. 2016). The airlock technique involves introducing a small air bubble after medication administration to create a seal, potentially reducing injection‐associated pain. However, further research is needed to ascertain its effectiveness and safety in clinical practice. Cold application, such as the application of a cold compress, has demonstrated promise in reducing bruising and discomfort at the injection site (Amaniyan et al. 2016; Varghese 2022). Cold application slows down local blood circulation, minimising the risk of bleeding and bruising, while also alleviating inflammation. However, standardisation of cold application techniques is essential to facilitate accurate comparisons and gain a comprehensive understanding of long‐term effects and patient satisfaction.

#### 6.1.4 Implications and the Need for Evidence‐Based Guidelines

Given the heterogeneity and limitations of existing studies, there is a pressing need for evidence‐based guidelines to optimise the injection technique for LMWH administration. The development of standardised protocols based on high‐quality RCTs will establish best practices, ensure consistency in clinical practice and evaluate its cost‐effectiveness and long‐term effects.

### 6.2 Strengths and Limitations

This scoping review has several strengths. It provides a broad overview of the existing literature on minimising adverse effects of subcutaneous LMWH injections. Multiple databases were searched to identify relevant studies. Furthermore, both objective outcomes and patient‐reported outcomes were considered, providing a comprehensive evaluation. The findings offer valuable insights into potential techniques that may effectively reduce injection pain and bruising.

However, it is important to acknowledge certain limitations. Firstly, the review only included studies published in the English language, which may have resulted in the exclusion of relevant non‐English studies. Additionally, there was considerable heterogeneity across the included studies in terms of interventions, comparators, outcomes and assessment tools, thereby precluding a quantitative synthesis. Furthermore, the quality of the included studies was not critically appraised, and the review solely provides a descriptive analysis without a formal quality assessment. Hence, cautious interpretation is necessary due to the inherent heterogeneity and the early stage of research in this particular area.

### 6.3 Future Research

Future research should focus on comparing different injection techniques, durations, waiting times and postinjection care practices. Additionally, investigations into the stability of medication at the injection site, drug distribution patterns and their impact on patient satisfaction and adherence are warranted. By establishing evidence‐based guidelines and conducting further research, healthcare professionals can enhance the safety and efficacy of LMWH administration, improve patient experience and optimise clinical practice.

## 7 Conclusions

This scoping review provides a summary of the existing literature on interventions aimed at minimising pain, bruising and other adverse effects associated with subcutaneous LMWH injections. Further investigation is warranted to standardise techniques for cold application and pressure, taking into account patient demographics and cost‐effectiveness considerations, in order to establish universally applicable postinjection care practices for LMWH administration. Based on the available studies, healthcare professionals may consider implementing interventions such as abdominal injections, slower injections and airlock techniques to help minimise pain and bruising associated with LMWH injections.

By systematically mapping out and analysing the existing research, this review provides healthcare professionals with evidence‐based insights into the most effective practices for reducing adverse effects such as bruising and bleeding, which are common complications of LMWH injections. The identification of potential strategies, such as using the abdominal site and slower injection rates, may help reduce adverse effects and improve patient outcomes in CVD management, enhancing the quality of care and ensuring patient safety in clinical settings. Consequently, this review serves as a valuable resource for clinicians and nurses, offering practical guidelines that can be incorporated into routine care to minimise the risks associated with LMWH injections in patients with CVD.

## Author Contributions

A.K.C.W., R.Y.K.C., Y.N. and J.M.: conceptualisation and methodology. A.K.C.W., R.Y.K.C., Y.N. and J.M.: data curation and writing – original draft preparation. A.K.C.W. and J.M.: writing – reviewing and editing. All authors contributed to, reviewed and approved the manuscript.

## Ethics Statement

The authors have checked to make sure that our submission conforms as applicable to the Journal's statistical guidelines as described here.

## Conflicts of Interest

The authors declare no conflicts of interest.

## Peer Review

The peer review history for this article is available at https://www.webofscience.com/api/gateway/wos/peer‐review/10.1111/jan.16475.

## Supporting information


Data S1.

## Data Availability

Data sharing not applicable—no new data generated.
